# Evaluation of bacterial safety approaches of platelet blood concentrates: bacterial screening and pathogen reduction

**DOI:** 10.3389/fmed.2024.1325602

**Published:** 2024-04-05

**Authors:** Mohammad Reza Rezvany, Amin Moradi Hasan-Abad, Ali Sobhani-Nasab, Mohammad Ali Esmaili

**Affiliations:** ^1^Department of Hematology, Faculty of Allied Medicine, Iran University of Medical Sciences, Tehran, Iran; ^2^BioClinicum, Department of Oncology-Pathology, Karolinska Institute, Stockholm, Sweden; ^3^Pediatrics Growth and Development Research Center, Institute of Endocrinology and Metabolism, Iran University of Medical Sciences, Tehran, Iran; ^4^Autoimmune Diseases Research Center, Shahid Beheshti Hospital, Kashan University of Medical Sciences, Kashan, Iran; ^5^Physiology Research Center, Institute for Basic Sciences, Kashan University of Medical Sciences, Kashan, Iran; ^6^Department of Laboratory Sciences, Sirjan School of Medical Sciences, Sirjan, Iran

**Keywords:** platelet transfusion, the safety of platelet products, prevention strategies, pathogen reduction systems, bacterial contamination screening

## Abstract

This mini-review analyzed two approaches to screening bacterial contamination and utilizing pathogen reduction technology (PRT) for Platelet concentrates (PCs). While the culture-based method is still considered the gold standard for detecting bacterial contamination in PCs, efforts in the past two decades to minimize transfusion-transmitted bacterial infections (TTBIs) have been insufficient to eliminate this infectious threat. PRTs have emerged as a crucial tool to enhance safety and mitigate these risks. The evidence suggests that the screening strategy for bacterial contamination is more successful in ensuring PC quality, decreasing the necessity for frequent transfusions, and improving resistance to platelet transfusion. Alternatively, the PRT approach is superior regarding PC safety. However, both methods are equally effective in managing bleeding. In conclusion, PRT can become a more prevalent means of safety for PCs compared to culture-based approaches and will soon comprehensively surpass culture-based bacterial contamination detection methods.

## 1 Introduction

More than 100 million blood units are transfused annually, making blood transfusion one of the most common hospital procedures (1). Among these, platelet concentrate (PC) transfusion is vital in various hematological and oncological diseases. Studies show 9–30% of intensive care unit (ICU) patients receive platelet transfusions (2, 3).

Infections caused by bacteria entering the patient’s body, transfusion-transmitted bacterial infections (TTBIs), remain a significant cause of mortality and morbidity. According to the US Food and Drug Administration (US FDA) report, infections caused by bacterial contamination between 2012 and 2016 are the third cause of mortality caused by blood transfusion (4). Compared to viral infection, bacterial infection occurs 100 to 1,000 times more.

The testing for bacterial contamination in PCs is typically done through platelet fraction sampling, as PCs are more susceptible to bacterial contamination than other products (4, 5). Bacterial contamination can range from a very low titer to as high as 10^10^ colony-forming units (CFUs) in each bag (6, 7).

While culture-based methods are still considered the gold standard in detecting bacterial contamination in PCs, they do not eliminate the risk of transmission of TTBIs (5, 8). There are two ways to increase the safety of PCs: pathogen reduction (PR) and bacterial screening (7, 9). Presently, three commonly used technologies—INTERCEPT (confirmed by the US FDA), Mirasol, and THERAFLEX—employ exposure to ultraviolet (UV) light to reduce the risk of various pathogens (10).

In a previous review, we evaluated screening methods for bacterial contamination in PCs (5). This mini-review is based on the PubMed database to analyze the advantages and disadvantages of various PRTs and bacterial screening. All articles were collected until October 2023. This review focuses on identifying bacterial contamination in PCs, as traditional tests involve sampling the platelet portion.

## 2 Bacterial contaminations

Platelet concentrates (PCs) were first introduced as a therapeutic product in the 1960s and have since become a crucial component in treating thrombocytopenia, particularly in cases caused by chemotherapy (11). PCs include single donor platelets (SDPs) and whole blood platelets (WBPs). There are two methods for preparing whole blood platelets (WBPs): Buffy coat (BC) and platelet-rich plasma (PRP).

The primary contamination source is bacteria from the skin’s normal flora due to inadequate sterilization of equipment and surfaces. PCs may contain Gram-positive and Gram-negative bacteria (5, 12). Notably, *Staphylococcus epidermidis*, a Gram-positive bacterium, is the most frequently isolated organism in PCs (5).

PCs now have a 5-day shelf life, instead of 7, to comply with US FDA recommendations and prevent bacterial contamination (12). Some countries, such as Japan, have further reduced the lifespan of PCs from 5 days to 3 days to reduce bacterial contamination (13). However, it is essential to note that bacterial contamination is only sometimes detectable before PCs are injected. PCs are the most susceptible to infection and sepsis of all blood products, primarily because they are stored at room temperature (RT). However, PRT is an effective method for extending the shelf life of PCs by up to 7 days (2).

Storage PCs at a cold temperature (1–6°C) can also be effective in reducing the growth of bacteria, maintaining platelet function (14), and, at the same time, increasing their lifespan (11). Most studies in the last two decades found platelet concentrates cold storage (CS) is beneficial (15). Some studies have shown that CS can have harmful and even irreversible effects on their morphology and function (16, 17). There is also some concern about whether PRT can be used on CS platelets without negatively affecting their therapeutic effectiveness (18). More research is needed to determine the effectiveness of CS platelets in treating acute bleeding despite their superiority over RT platelets (19, 20). The US FDA recently approved a 14-day shelf life of PCs to treat active bleeding only when RT platelets are unavailable (21).

The first prevention strategy involves screening and selecting donors based on their medical condition and asking about any recent antibiotic treatments (22). Immunological procedures to prevent transfusion-transmitted bacterial infections (TTBIs) begin with careful donor screening and blood testing for known pathogens (23). However, this approach cannot detect asymptomatic bacteremia (22, 24). Another effective method is to remove a small amount of blood (around 10–20 mL) at the beginning of the collection process, which can significantly reduce the risk of bacterial contamination, especially from gram-positive bacteria, although it cannot eliminate it. The diversion method is a standard for preventing bacterial contamination (24).

Two other significant strategies include bacterial screening methods and pathogen reduction technologies (PRTs) (2). Table 1 provides a detailed comparison of these strategies.

**Table 1:** Comparison of advantages and disadvantages of two strategies of bacterial screening and pathogen reduction (PR) of platelet concentrates (PCs).

	Bacterial screening	Pathogen reduction	References
Non-inferiority^a^ of PCs	More	Less	(10, 25–27)
Platelet Refractoriness	Less	More	(28)
False-positive or -negative results	Yes	No	(29)
Logistical issues	No/Yes (for culture-based methods)	Yes	(29)
Timing of treatment	Significant	Critical	(29)
Shelf life of PCs	3–7 days	5–7 days	(2)
Approach	Non-proactive	Proactive	(2)
Safety level	Variable: Low to high (for culture-based methods)	The highest	(29)

^a^Non-inferiority relates to platelet metabolic activity, physiology, function, and storage lesions *in vitro*.

## 3 Discussion

### 3.1 Bacterial screening methods of platelet concentrates

While the reported frequency of bacterial contamination ranges from 1 in 750 to 1 in 2,500 blood units, both transfusion-transmitted bacterial infections (TTBIs) and sepsis transfusion reactions (STRs) are often underreported and can seriously threaten patients (2). One factor contributing to this issue is that many centers only perform aerobic blood cultures, which cannot detect anaerobic bacterial contamination (30). Additionally, some hospitals fail to quarantine PCs for the recommended 12 to 24 h, which increases the risk of STRs. However, even a 24-h quarantine may not eliminate slow-growing or non-multiplying bacteria in storage conditions.

The second critical strategy in preventing the occurrence of bacterial, PC screening methods, can be divided into two categories: culture-based and non-culture-based.

First, non-culture-based methods offer the crucial advantage of being time-effective. Although culture-based methods will remain the gold standard, the significance of rapid methods for screening for bacterial contamination has increased. Rapid new methods provide powerful tools for improving the bacterial safety of blood components (6). The analytical sensitivity between the different detection methods ranged between 50 and 100,000 CFU/mL. The sample volume these testing systems use varies between 0.5 and 1.0 mL of PCs (31). Generally, non-culture-based methods are classified into low-sensitivity and high-sensitivity subgroups.

The high-sensitivity subset is composed of nuclear polymerase reaction (PCR) with a sensitivity of 10–10^3^ colony forming unit (CFU) per milliliter of platelet concentrate (ml PC) and flows cytometry with a sensitivity of 10–10^3^ CFU (mL PC) (32). Nonetheless, implementing non-culture techniques is complicated and costly regardless of the challenges of detecting bacterial contamination during the first few days of PC storage life.

There are several low-sensitivity methods to detect bacterial contamination in PCs. These methods include gram staining, acridine orange, glucose consumption, pH measurement, pan genera detection (PGD), and bio-responsive polymers. Gram staining and acridine orange have similar sensitivity levels (10^5^–10^6^ CFU/mL PC) but require significant time and expertise to perform accurately. Moreover, their favorable detection rates are low (33). Glucose consumption and pH measurement methods are not commonly used in clinical settings due to their high rate of false positives (34). However, using these methods shortly before injection can enhance PCs’ safety (35). The PGD method is a qualitative immunological technique that detects lipopolysaccharide (LPS) in gram-negative bacteria and lipoteichoic acid in Gram-positive bacteria. Bio-responsive polymers use enzyme reactions to detect bacterial contamination (36). Of all the methods listed, only the PGD approach is approved by the US FDA. It is used to ensure the safety of blood distribution points and for quality control measurements. This test extends the lifespan of PCs from 5 days to 7 days by measuring them within 24 h of injection on day 6 or 7 (or both) (2, 6).

Non-culture-based methods are generally known for having high false positive results and needing to be more laborious and costly (2). The visual inspection method can effectively identify bacterial contamination in its place (37). Sample collection should occur in the final 48 h of PC storage or just before product injection due to their lower sensitivity for non-culture-based methods. The key benefit of these methods is their ability to produce rapid results before PC injection (31).

Second, culture-based methods are currently considered the most reliable way to detect bacterial contamination. These methods can be used to check for contamination during the storage period of PCs or shortly before they are given to patients. However, waiting until before injection may be too late to prevent contamination. It is recommended to check for contamination on the third or fourth day of the platelet product’s storage period (38). However, these methods require a large sample and an incubation time of more than 24 h (39). The BacT/ALERT and eBDS systems have been approved by the FDA.

Culture-based systems generally have a high sensitivity and can detect 1–10 CFU per ml. However, their sensitivity may need to be revised and can vary depending on the age of the product being tested (8). Most culture-based systems may not be effective in detecting biofilm-producing bacteria (6, 13). Bacterial culture quality control in PCs is primarily carried out through culture-based methods. However, these methods are time-consuming and are not considered rapid screening methods. Research has shown that sepsis caused by PCs cannot be entirely prevented, regardless of the age of the product (37).

### 3.2 Pathogen reduction technologies

Pathogen reduction (PR) is a highly effective third strategy that has proven particularly useful in reducing bacterial contamination in PCs. PR technologies (PRTs) that employ ultraviolet (UV) rays are commonly used in blood products to eliminate contamination and prevent TTBIs. The irreversible prevention of DNA replication and RNA transcription by UV rays is highly effective in targeting viruses, bacteria, parasites, protozoa, and other nucleated cells, such as leukocytes (2). In addition, PRT is a proactive approach compared to donor screening strategies and PC screening (9).

PRT can effectively prolong the shelf life of PCs (to 7 days). Platelet activation and storage lesions (PSLs) are inherent to PR technologies. Further studies are necessary to improve the safety and efficacy of platelet recovery treatments, as these lesions can be large enough to affect post-transfusion platelet survival. A platelet additive solution (PAS) has not entirely solved these problems (40). Other studies have revealed that PR’s effect on platelet function during storage of PCs is minimal depending on time, particularly in the last days (5th and later) and technology employed. However, PRT treatment may require more injections at shorter intervals and may be associated with increased platelet resistance and alloimmunization (28). On the other hand, most studies indicate that PRT has the necessary safety standards for injection to patients (41–46).

Despite the challenge posed by the cost of these technologies (47), data have shown that they can be cost-effective compared to other blood safety interventions (48). Results of a review study revealed that PCs have the highest safety standards in countries such as Belgium, Switzerland, and France, where PR is performed. However, there are logistical challenges (centralized vs. peripherally localized platelet product preparation sites, hospital-based vs. regionally or nationally organized blood services, etc.), potential risks of pyrogenic compounds, insufficient killing of sporulating and biofilm-producing bacteria, time of treatment, as well as additional costs that need to be considered. Although some of these challenges, such as additional cost and logistical issues, may also be seen in other strategies, especially culture-based methods (29). Finally, the anticipated benefits of PRT should be carefully evaluated before making a decision (49).

Various factors, including the timing of treatment (50) and the entity of bacteria present, such as growth speed (fast-growing vs. slow-growing) (51) or sporulation, can directly affect the effectiveness of PRT. If PR is delayed after blood collection, bacteria may grow and reach high levels, potentially breaking through the system function (52). For example, A study showed that the delay of treatment with Mirasol from 26 to 32 h post-WB collection affects the efficiency of PR for *Klebsiella pneumoniae* strains (53). Following PR, endospore bacteria such as *Bacillus subtilis* and *Bacillus cereus* showed PC growth and could be detected using RT-PCR and automated culture (54). Furthermore, post-manufacturing contamination of pathogen-reduced PLT may have been caused by environmental sources or inherent/acquired bag defects (55). In contrast to late bacterial culture, treating PC with PR methods early is essential to avoid high bacterial loads (29, 56). It has been suggested that in cases where PR cannot be performed immediately after preparation, a combination of PR technology with a rapid bacterial screen test on the 4th or 5th day after donation may offer a potential solution further to mitigate the risk of bacterial transmission by transfusion (56).

**Figure 1:**
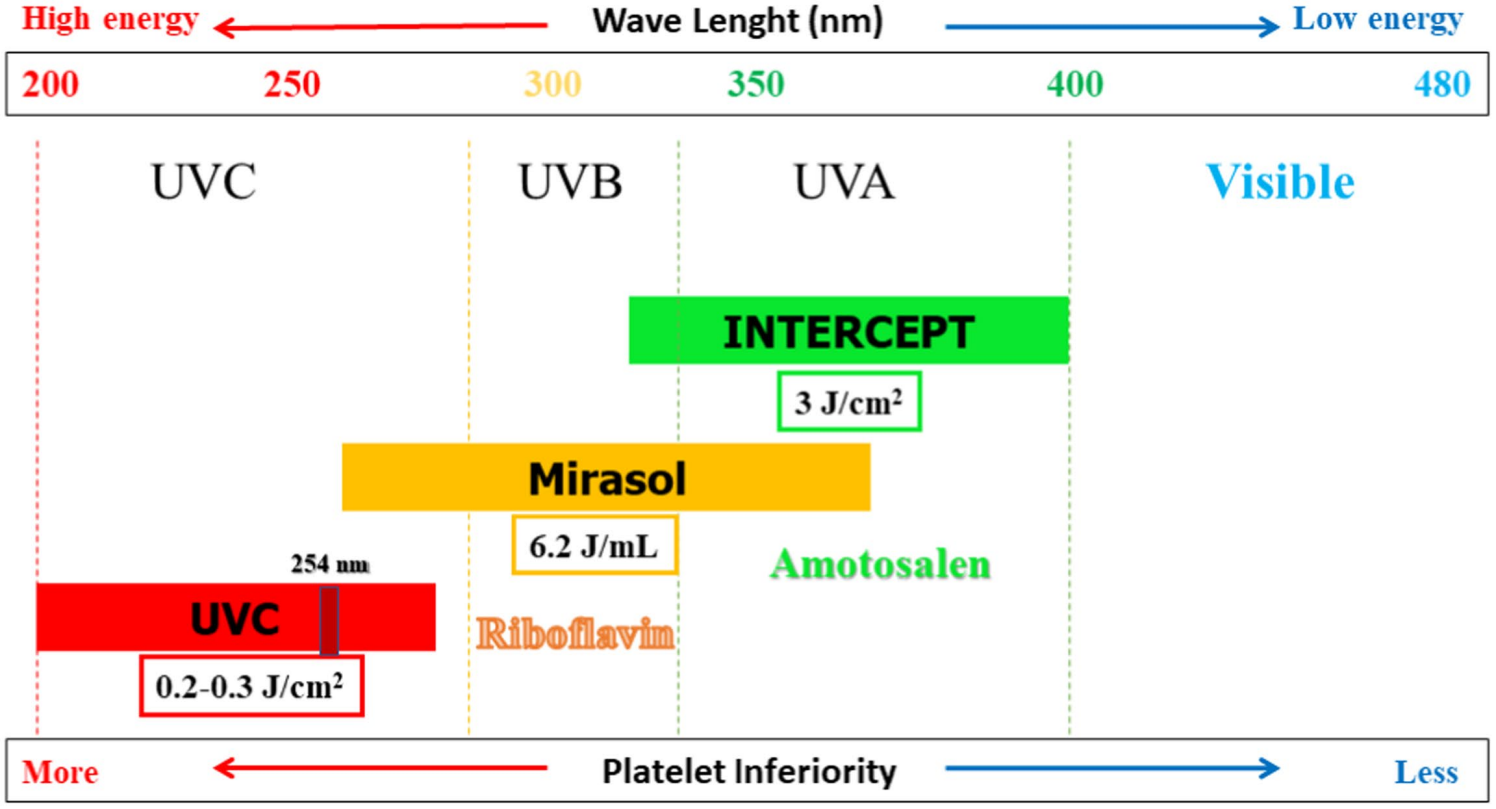
Pathogen reduction technologies (PRTs) compatible with platelet concentrates (PCs): Dose, wavelength, energy level, and photosensitizer are shown.

Since 1990, several techniques have been developed to reduce pathogens that may be chemically present in PCs. Table 2 provides an overview of the characteristics of three common PRTs that are compatible with platelets (2, 55). These technologies differ regarding the UV wavelengths, doses, energy levels, and photosensitive compounds employed (Figure 1). Typically, a lower wavelength accompanied by a higher energy level is more effective in causing damage to the pathogen cells. The three technologies are INTERCEPT, Mirasol, and THERAFLEX. In the case of the first two technologies, a photosensitive compound is added to the PCs before UV exposure. For THERAFLEX, short-wavelength UV is employed (23).

**Table 2:** An overview of the comparison of the features of three leading pathogen reduction technologies (PRTs) (2, 55, 57).

Technology	INTERCEPT	Mirasol	THERAFLEX
Manufacturer	Cerus Corporation, Concord, CA, United States	Terumo BCT, Lakewood, CO, United States	Macopharma, Mouvaux, France
UV wavelength and dose	UVA, 320–400 nm, 3 J/cm^2^	UVB/UVA/UVC (100%/60%/20%), 265–370 nm, 6.2 J/mL	UVC, 200–280 nm, 0.2–0.3 J/cm^2^
Photosensitizer	Amotosalen (S-59)	Riboflavin (B2 vitamin)	None
Adduct	S-59 intercalation inter-and intra-strand cross-linking	8-oxodG strand breaks	6,4 thymine dimers
Additional steps	Filtration, post illumination	None	None
How it prevents DNA proliferation	Intercalation in helical regions	Oxido-reductive damage	Pyrimidine dimerization
Typical consequences	Amotosalen remains on platelets due to lipid binding	Increases platelet anaerobic metabolism rates	“Primes” platelets for activation due to reduced disulfide bonds
Pathogens targeted	Bacteria (Gram-positive and Gram-negative), viruses (enveloped and non-enveloped), parasites	Bacteria (Gram-positive and Gram-negative), viruses (enveloped and non-enveloped), parasites	Bacteria (Gram-positive and Gram-negative), viruses (enveloped and non-enveloped), parasites
Degree of reduction of *Staphylococcus epidermidis* in Log	≥6.6	4.2	4.8
Formation of bacterial biofilm	Significant	Significant	Not significant
Availability	Commercial	Commercial	Not yet in routine use
Maximum approved storage	5–7 days (platelets)	7 days (platelets)	5 days (platelets)
	2 years at ≤30°C (plasma)	2 years at ≤30°C (plasma)	2 years at ≤30°C (plasma)
CE mark approval	CE class III	CE class IIB	class IIB
FDA approval for platelets	Yes	No (Phase III Clinical Trial in the United States)	No

CE, European Conformity; US FDA, US Food and Drug Administration; UV, ultraviolet.

INTERCEPT technology, developed by Cerus Corporation based in Concord, CA, United States, has received FDA approval for its use of Amotosalen as a photosensitizing compound in the process of exposing PCs to ultraviolet A (UV A) light ranging from 320 to 400 nm. To minimize the potentially toxic effects, we have a process to remove residual Amotosalen safely. Any remaining Amotosalen (synthetic psoralen) is then safely removed through absorption. This process is typically completed within 24 h of platelet donation within the blood collection department (2, 55). The INTERCEPT system was not 100% effective for high concentrations of certain *Klebsiella pneumoniae* strains or spore-forming *Bacillus cereus*. Like other PR systems, this technology has limitations for fast-growing and spore-forming bacteria (56). Data has demonstrated that the use of INTERCEPT technology does not harm the metabolism or function of CS platelets (30). Using PCs containing triple sugar (TS) and platelet additive solution III or IIIM (PAS-III or PAS-IIIM) is relatively safe (58). INTERCEPT technology is more effective than Mirasol technology in maintaining the quality of platelets *in vitro*, and 7 days of storage are achieved based on quality criteria using INTERCEPT technology (59). However, there is also the possibility of microbial resistance (60).

In Mirasol technology (Terumo BCT, Lakewood, CO, United States), riboflavin (vitamin B2) is used as a light-sensitive compound, followed by ultraviolet B/A/C spectrum (265–370 nm). Unlike Amotosalen, there is no need to remove residual riboflavin. Its action mechanism strongly depends on reactive oxygen species, and guanine bases are selectively targeted. This technology, which received European conformity Class IIB (CE class IIB) in 2007, is used in over 20 countries (2, 55, 61). It is currently in a phase III clinical trial in the United States (47). In this technology, riboflavin is mixed with PC and then exposed to UV light for less than 10 min (62). Studies show the impact of this technology on different bacterial species (41). Based on plate count, the PR capability of the Intercept method is greater than that of the Mirasol method (63). Platelet loss level is significantly higher in the INTERCEPT units than in the Mirasol units. Additionally, the harmful impact and level of platelet loss are more elevated in WBPs than in single SDPs (59). However, this technology is only partially effective in deactivating biofilm-producing species such as *Staphylococcus epidermidis* (64).

The THERAFLEX technology, developed by Macopharma in Mouvaux, France, does not require a photosensitive compound for PCs. Instead, it uses short wavelength UV C light (254 nm) in PCs with agitation. This light gets absorbed by nucleic acids, forming pyrimidine dimers that stop nucleic acid transcripts’ elongation. 2009, this technology was awarded the European Conformity of class IIB (CE class IIB). Using a wavelength of 265 nm effectively inactivated bacteria, especially *Staphylococcus aureus* and *Bacillus cereus* (8). Several studies have assessed the impact and effectiveness of THERAFLEX technology in PCs (25). According to the available data, bacterial biofilm formation is not a significant concern in the context of the THERAFLEX UV-Platelets procedure. Proper pre-treatment with PI is crucial in this process, followed by the transfer of platelets to the illumination bag. Biofilms can provide a protective layer to encapsulated bacteria, making them less susceptible to inactivation by reducing the penetration of UV light or photochemical (64).

In conclusion, PRTs can be highly efficient in eliminating various pathogens. However, they may need to be more practical, completely inactivating pathogens in PCs. PRTs can impact platelets’ activation, function, and survival and may encounter Microbic resistance. The platelet additive solution (PAS) is a partial approach to solving them. Through extensive research, identifying and resolving preclinical and clinical shortcomings, and optimizing their advantages, PRTs will soon comprehensively surpass culture-based bacterial contamination detection methods. An important reason is that the maximum safety of PCs in using PRT has been shown in countries such as France, Switzerland, and Belgium compared to Germany, Italy, Bulgaria, and Poland without mandatory use (29). To promote widespread adoption, reinforcing preclinical and clinical safety standards of PRTs is imperative.

## Author contributions

MR: Writing – original draft, Validation. AM: Writing – original draft. AS-N: Writing – original draft. ME: Writing – original draft, Supervision, Writing – review & editing.
